# *Treponema denticola* TroR is a manganese- and iron-dependent transcriptional repressor

**DOI:** 10.1111/j.1365-2958.2008.06418.x

**Published:** 2008-08-29

**Authors:** Paul J Brett, Mary N Burtnick, J Christopher Fenno, Frank C Gherardini

**Affiliations:** 1 Laboratory of Zoonotic Pathogens, Rocky Mountain Laboratories, NIAID, NIHHamilton, MT 59840, USA; 2 Biologic and Materials Sciences, School of Dentistry, University of MichiganAnn Arbor, MI 48109, USA

## Abstract

*Treponema denticola* harbours a genetic locus with significant homology to most of the previously characterized *Treponema pallidum tro* operon. Within this locus are five genes (*troABCDR*) encoding for the components of an ATP-binding cassette cation-transport system (*troABCD*) and a DtxR-like transcriptional regulator (*troR*). In addition, a σ^70^-like promoter and an 18 bp region of dyad symmetry were identified upstream of the *troA* start codon. This putative operator sequence demonstrated similarity to the *T. pallidum* TroR (TroR_Tp_) binding sequence; however, the position of this motif with respect to the predicted *tro* promoters differed. Interestingly, unlike the *T. pallidum* orthologue, *T. denticola* TroR (TroR_Td_) possesses a C-terminal Src homology 3-like domain commonly associated with DtxR family members. In the present study, we show that TroR_Td_ is a manganese- and iron-dependent transcriptional repressor using *Escherichia coli* reporter constructs and in *T. denticola*. In addition, we demonstrate that although TroR_Td_ possessing various C-terminal deletions maintain metal-sensing capacities, these truncated proteins exhibit reduced repressor activities in comparison with full-length TroR_Td_. Based upon these findings, we propose that TroR_Td_ represents a novel member of the DtxR family of transcriptional regulators and is likely to play an important role in regulating both manganese and iron homeostases in this spirochaete.

## Introduction

*Treponema denticola*, a member of the ‘red complex’, is a motile, asaccharolytic, anaerobic spirochaete that is common to the normal oral flora of humans (Socransky *et al*., 1998; Sela, 2001). Studies indicate that it represents one of approximately 60 treponemal species associated with subgingival plaque (Paster *et al*., 2001). The organism is thought to contribute significantly to the development of a number of acute and chronic periodontal diseases, including severe periodontitis, a polymicrobial infection of gingival tissues that if left untreated can lead to bone resorbtion and tooth loss (Loesche and Grossman, 2001). Although not generally considered to be life-threatening, the economic impact of periodontal disease in adult populations is substantial (Satcher, 2000; Loesche and Grossman, 2001). *T. denticola* has been shown to express a variety of adhesins, proteases, cytolysins and immunomodulatory factors (Sela, 2001). Many of these are predicted to play important role(s) in the pathogenesis of diseases caused by this organism.

In order to successfully colonize human hosts, bacteria must be able to acquire nutrients and overcome the constant pressure of host defences. Because metal ions are important for many biological processes, the ability of bacteria such as *T. denticola* to obtain essential metals from the environmental niches in which they reside is critical for their survival (Outten and O'Halloran, 2001; Blencowe and Morby, 2003; Cornelissen, 2003; Mulrooney and Hausinger, 2003; Rensing and Grass, 2003). Although human tissues, fluids and secretions are seemingly rich in nutrients, many elements, including Fe^2+^, Zn^2+^ and Mn^2+^, are often not freely available for use by bacteria (Ratledge and Dover, 2000; Kaplan, 2002; Braun, 2003). In order to cope with this problem, microorganisms have evolved both passive and active mechanisms for acquiring metals from these environments (Ferguson and Deisenhofer, 2004). In general, passive mechanisms are associated with high rates of transport and low metal-binding affinities, while active mechanisms show slower transport kinetics with higher binding affinities (Nikaido and Saier, 1992; Brown and Holden, 2002; Cornelissen, 2003; Faraldo-Gomez and Sansom, 2003). With regard to iron acquisition, *T. denticola* has been shown to bind both lactoferrin and haemin but does not produce siderophores (Scott *et al*., 1993; Chu *et al*., 1994; Xu *et al*., 2001; Xu and Kolodrubetz, 2002). At present, the mechanisms by which spirochaetes such as *T. denticola* obtain other essential metals from host environments remains poorly understood.

Consistent with the predicted metabolic diversity of *T. denticola*, genome analyses indicate the presence of a variety of putative metal transport-related loci (Seshadri *et al*., 2004; Gherardini *et al*., 2006). In addition to possessing homologues of *Treponema pallidum* ZnuABC and TP0972, *T. denticola* appears to possess eight ATP-binding cassette (ABC)-type transporters specifically devoted to the transport of Fe^2+^, implicating the importance of this metal to the organism (Seshadri *et al*., 2004; Gherardini *et al*., 2006; Desrosiers *et al*., 2007). Particularly interesting, however, is the presence of a Tro ABC-transport system similar to that identified in *T. pallidum* (Hardham *et al*., 1997; Gherardini *et al*., 2006). Comparative analyses of the *T. pallidum* and *T. denticola tro* operons have demonstrated that the genetic organization of the *troABCDR* locus in both species is identical and that a high degree of sequence similarity exists between the various gene products at the amino acid level (Gherardini *et al*., 2006). In the present study we utilize a combination of molecular genetic and biochemical approaches to demonstrate that *T. denticola* TroR (TroR_Td_) is a cation-dependent transcriptional repressor possessing structural and biochemical properties distinct from its *T. pallidum* orthologue.

## Results

### Identification of the *tro* operon in *T. denticola*

**Fig. 1:**
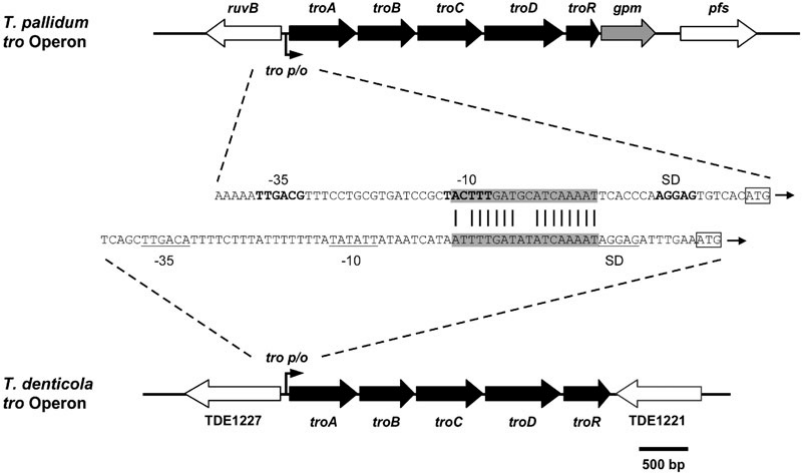
Genetic organization of the *T. pallidum* and the *T. denticola tro* operons and sequence alignment of the *T. pallidum* and *T. denticola tro* P/O regions located immediately upstream of the *troA* ORFs. *troA* is predicted to encode a periplasmic binding protein; *troB* is predicted to encode the ATP-binding component, while *troC* and *troD* are predicted to encode the membrane components of ABC transporters; *troR* is homologous to a number of genes encoding Gram-positive iron-activated repressor proteins including DtxR and SirR; *gpm* encodes the glycolytic pathway enzyme, phosphoglycerate mutase. The σ^70^-like promoters (−10 and −35 sequences) are indicated in bold (*T. pallidum*) or underlined (*T. denitcola*). The 18 bp operator motifs are highlighted in grey. Sequence identity between the operators is indicated by vertical lines. Putative ribosome binding sites are in bold (*T. pallidum*) or underlined (*T. denitcola*) and indicated by SD. The *troA* start codons are indicated by boxes.

Analysis of the *T. denticola* ATCC 35405 genome sequence revealed the presence of a genetic locus demonstrating significant similarity to the *T. pallidum tro* operon. In *T. denticola*, the *tro* operon consists of five open reading frames: *troA*, *troB*, *troC*, *troD* and *troR*. This operon is predicted to encode the components of an ABC cation-transport system composed of TroA, a solute-binding protein; TroB, an ATPase; TroC and TroD, cytoplasmic membrane permeases; and TroR, a metal-dependent transcriptional regulator (Hardham *et al*., 1997). In contrast to *T. pallidum*, however, *gpm* encoding the glycolytic pathway enzyme, phosphoglycerate mutase is absent and the genes flanking the operons are not conserved (Fig. 1). Results of comparative analyses of the *tro* operons from these two organisms are shown in Table 1. The *troABCD* orthologues are highly similar in both species; however, unlike *T. pallidum* TroR (TroR_Tp_), TroR_Td_ harbours a 70-amino-acid C-terminal extension.

**Table 1:** Pairwise comparisons of *T. denticola and T. pallidum* open reading frames.

	*T. denticola/T. pallidum*				
	
Open reading frame	Amino acids	Predicted mass	Predicted pI	% Identity	% Similarity
*troA*	312/308	34583/33570	5.58/6.21	54	73
*troB*	255/266	28572/29360	7.69/6.46	74	88
*troC*	304/298	32359/31540	10.14/10.26	61	79
*troD*	366/367	39760/38776	8.60/8.60	60	77
*troR*	222/153	25412/17122	9.15/8.47	55	74

Inspection of the region immediately upsteam of the *T. denticola troA* start codon revealed the presence of a σ^70^-like promoter sequence and an 18 bp region of dyad symmetry (Fig. 1). DNA sequence alignment of this region with that of *T. pallidum* revealed that 15/18 bp wasidentical to the previously described TroR_Tp_-binding sequence. Interestingly, the relative locations of the putative *T. denticola tro* promoter and operator (*tro*-P/O) sequences differ relative to the *T. pallidum tro*-P/O sequences (Fig. 1). While the *T. pallidum tro*-P/O sequences have been determined to be overlapping (Posey *et al*., 1999), the predicted *T. denticola tro*-P/O sequences are separated by 10 bp (Fig. 1).

### Bioinformatic analysis of TroR_Td_ and purification of TroR_Td_-6×His

**Fig. 2:**
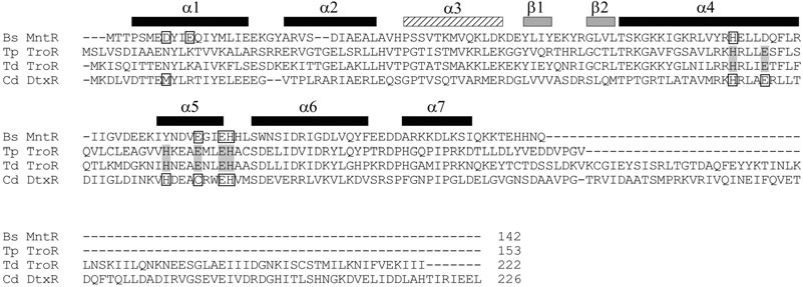
Primary sequence alignment of *B. subtilis* MntR (Bs MntR), *C. diptheriae* DtxR (Cd DtxR) and the *T. pallidum* (Tp) and *T. denticola* (Td) TroR proteins. The secondary structure of DtxR family metalloregulators is indicated above the sequence alignments: α-helices (α1, 2, 4, 5, 6) are shown in black, the DNA recognition helix (α3) is shown as a hatched line, β-strands (β1–2) are shown in grey and the proline-containing tether region (α7) is shown in black. The black boxes outline conserved metal co-ordination site residues in Bs MntR or Cd DtxR sequences. Putative TroR metal co-ordination site residues are highlighted in grey.

TroR_Td_ is predicted to be a metal-dependent transcriptional regulator based on homology to the DtxR family of metalloregulators. This group of transcriptional regulators contain several Fe-activated repressor proteins, including *Corynebacterium diptheriae* DtxR, *Mycobacterium tuberculosis* IdeR and *Streptomyces* spp.; DesR, the Mn^2+^-activated regulators TroR_Tp_ and *Bacillus subtilis* MntR; and the Fe- and Mn^2+^-activated repressors *Staphylococcus epidermidis* SirR and Group A *Streptococcus* MtsR (Hill *et al*., 1998; Bates *et al*., 2005; Pennella and Giedroc, 2005). An alignment of the primary amino acid sequences of TroR_Td_ with TroR_Tp_, DtxR (Cd DtxR) and MntR (Bs MntR) is shown in Fig. 2. In general, Fe-responsive repressors have N-terminal domains (∼126 amino acids) containing helix–turn–helix DNA binding motifs (α2α3) and two metal ion binding sites (D'Aquino and Ringe, 2003). A short proline-containing region (∼26 amino acids) links this domain to the C-terminal region (∼76 amino acids) (Brennan and Matthews, 1989; Qiu *et al*., 1995; Schiering *et al*., 1995; D'Aquino and Ringe, 2003). TroR_Td_ is predicted to have secondary structure similar to DtxR and to contain a comparable N-terminal DNA-binding domain followed by a helical dimerization domain. Additionally, similar to DtxR, TroR_Td_ appears to possess an extended C-terminal Src homology 3-like (SH3-like) domain that is absent from TroR_Tp_ and MntR.

Two distinct metal co-ordination sites have been identified in DtxR: the ancillary cation binding site (site 1:H79, E83, H98) is thought to stabilize dimer formation and allow metal ion binding at the primary site (site 2: M10 and C102, E105, H106), which is the putative metalloregulatory site that is required for metal ion activation (Qiu *et al*., 1995; Ding *et al*., 1996; Love *et al*., 2003; Pennella and Giedroc, 2005). In comparison with DtxR, MntR and TroR_Tp_, TroR_Td_ demonstrates a high degree of conservation at both sites 1 and 2 with 5/7, 4/7 and 7/7 identical residues respectively (Fig. 2, shaded amino acids). Notably, an important difference at site 2 in both TroR_Td_ and TroR_Tp_ is the presence of an asparagine (N10) corresponding to DtxR M10 and MntR D8. Additionally, the C102 residue of DtxR is substituted by E102 in MntR, TroR_Td_ and TroR_Tp_. All four proteins are predicted to have tether regions linking the N- and C-terminal regions of the proteins; this region is designated α7 in MntR and is proline-containing in DtxR and TroR_Td_ (Fig. 2). The biological function of the DtxR C-terminal SH3-like domain is unclear; however, it has been proposed to modulate repressor activation through interactions with site 1 residues, and to contribute two ligands (E170 and Q173) involved in metal ion binding (D'Aquino and Ringe, 2003; Love *et al*., 2003; Chou *et al*., 2004). These two residues do not appear to be conserved in TroR_Td_ (Fig. 2).

In order to obtain purified protein, *T. denticola troR* was cloned into the pBAD/HisA expression vector resulting in pPJB114. The His-tagged protein (TroR_Td_-6xHis) was overexpressed in *Escherichia coli*, producing a 26 kDa protein that localized to the insoluble fraction of the cell lysate. Following removal of soluble proteins, TroR_Td_ was extracted from the insoluble pellet with 0.2% Sarkosyl and purified with a Nickel-agarose affinity column as described in the *Experimental procedures* section. This procedure yielded TroR_Td_-6xHis that was purified to apparent homogeneity as determined by SDS-PAGE (data not shown) and was used for antibody production. Following numerous attempts using a variety of conditions, including those previously used to purify TroR_Tp_ (Posey *et al*., 1999), we were unable to obtain TroR_Td_ from the soluble fraction, and a minimum of 0.2% sarkosyl was required to obtain TroR_Td_ from the insoluble fraction. Because of the insolubility of recombinant TroR_Td_, we were unable to conduct *in vitro* DNA binding experiments.

### TroR_Td_ expression negatively regulates both *T. denticola* and *T. pallidum tro* promoter activity

**Fig. 3:**
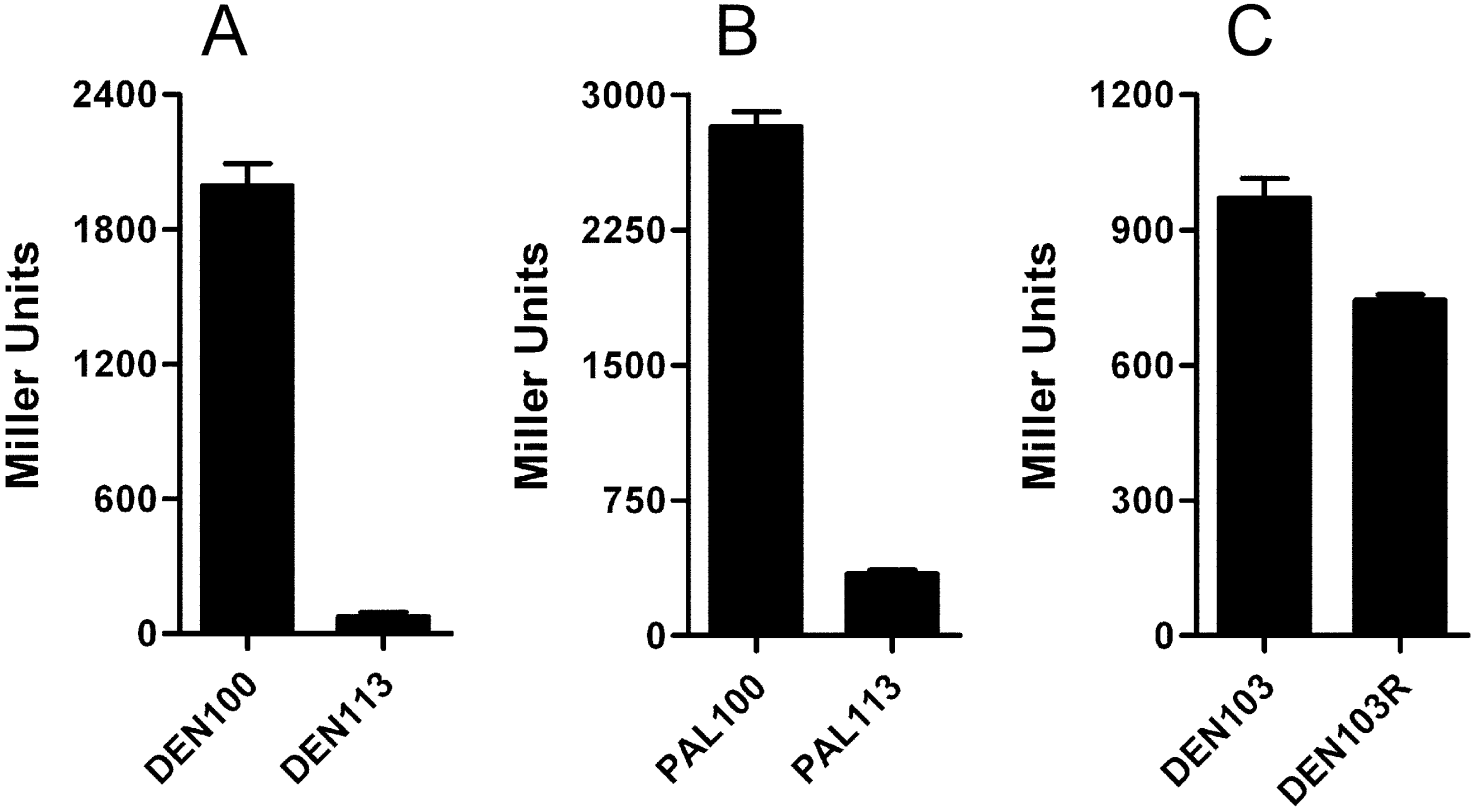
TroR_Td_ expression negatively regulates both *T. denticola* and *T. pallidum tro* promoter activity. β-Galactosidase activity was measured from: (A) strains DEN100 and DEN113 harbouring pTDE100 (*T. denticola tro*P/O-*lacZ* reporter) and either pBAD/HisA (control) or pPJB113 (expresses TroR_Td_); (B) strains PAL100 and PAL113 harbouring pPAL100 (*T. pallidum tro*P/O-*lacZ* reporter) and either pBAD/HisA (control) or pPJB113 (expresses TroR_Td_); or (C) strains DEN103 and DEN103R harbouring pTDE103 (*T. denticola tro*P/O-*lacZ* reporter with a disrupted operator region) and either pBAD/HisA (control) or pPJB113 (expresses TroR_Td_). Cultures were grown aerobically for 12 h in LBL media and β-galactosidase activity was determined as described by Miller (1972). Results represent the means and standard deviations of three independent experiments.

Studies by Posey *et al*. (1999) have previously demonstrated that purified TroR_Tp_ can bind to the *tro*-P/O region in a Mn^2+^-dependent manner. To assess the ability of TroR_Td_ to bind to and affect transcription from the *tro*-P/O region, we employed *lacZ*-transcriptional fusion analyses in *E. coli*. The *tro*-P/O regions from both *T. denticola* and *T. pallidum* were cloned into pPBMB101, generating *lacZ* reporter constructs, pTDE100 and pTPA100 respectively. β-Galactosidase activity was measured in *E. coli* TOP10 cells harbouring pTDE100 or pTPA100 in the presence of either pPJB113 (expresses TroR_Td_) or pBAD/HisA (vector control). Immunoblot analysis of *E. coli* lysates showed that TroR_Td_ was expressed from pPJB113, but not from the empty vector control (data not shown). Results demonstrated that β-galactosidase activity from strains DEN100 (harbouring pTDE100 and pBAD/HisA) and PAL100 (harbouring pPAL100 and pBAD/HisA) was high in the absence of TroR_Td_ (Fig. 3A and B). In contrast, when TroR was expressed, β-galactosidase activity decreased significantly in strains DEN113 (harbouring pTDE100 and pPJB113; 96% repression) and PAL113 (harbouring pPAL100 and pPJB113; 88% repression), indicating that TroR_Td_ exerted a negative regulatory effect on both of the *tro* promoters (Fig. 3A and B). These results are consistent with previous transcriptional fusion studies involving TroR_Tp_ (Posey *et al*., 1999) and suggest that TroR_Td_ acts as a repressor of the *tro* operons.

To confirm the importance of the putative *T. denticola tro* operator sequence for TroR_Td_ binding, this sequence was mutated and assessed using a *lacZ* reporter fusion. Plasmid pTDE103 harbouring the *T. denticola tro*-P/O was constructed with four base changes in the putative operator sequence (A**GC**TT**C**ATAT**T**TCAAAAT, changes in bold, Table 2). Results of β-galactosidase assays using strains DEN103 and DEN103R demonstrated that promoter activity from pTDE103 decreased by only 23% in the presence of TroR (Fig. 3C). Additionally, the β-galactosidase activity from pTDE103 was approximately twofold lower than pTDE100 (Fig. 3A and C), suggesting that changes in the operator may affect other factors involved in transcription from the *tro*-P/O region. These findings further support that TroR_Td_ is a negative regulator of the *tro* operon.

**Table 2:** PCR primers, complementary oligonucleotides and qRT-PCR primers and probes used in this study.

PCR primers	
OriCm-F1	5′-GATGCTAGATCTTCGAATTTCTGCCATTCATCCGC-3′
OriCm-R1	5′-GATGCTCTGCAGACTAGTCGACCCGGGATCCTCTAGAAATATTTTATCTGATTAATAAGATG-3′
LacZ-F2	5′-GATGCTCTGCAGCTCGAGTTCACACAGGAAACAGCTATGATAGATCCCGTCGTTTTACAACG-3′
LacZ-R2	5′-GATGCTAGATCTTACTCAGGAGAGCGTTCACCG-3′
Km-FNh	5′-CCCAACGGTCTCACTAGAGCGAACCGGAATTGCCAGCTG-3′
Km-RNc	5′-CCCAACGGTCTCACATGCTCAGAAGAACTCGTCAAGAAG-3′
TdtroR-F2	5′-CCCAACCGTCTCACATGAAAATCTCGCAAATTACAACCG-3′
TdtroR-R3	5′-CCCAACCGTCTCAAGCTTATTATATTCCGCATTTTACTTTATCTAAACTTG-3′
TdtroR-RA	5′-CCCAACCGTCTCAAGCTTACTAAATAATAATTTTTTCGACAAATATATTTTTTAAAATCATCG-3′
TdtroR-RB	5′-CCCAACCGTCTCAAGCTTATTAATCTGTACATGTATATTCCTTTTG-3′
TdtroR-RC	5′-CCCAACCGTCTCAAGCTTATTATCTGGGTATCATTGCTCCATGGG-3′
TdtroR-RD	5′-CCCAACCGTCTCAAGCTTATTATTTAGGATGTCCTAAATATTTGTC-3′
TdtroR-RH2	5′-CCCAACCGTCTCAAGCTTATTAATGATGATGATGATGATGAATAATAATTTTTTCGACAAATATATTTTTTAAAATCATCG
Complementary oligonucleotides	
Tdtrop-A	5′-GATCGGGATTCAGCTTGACATTTTCTTTATTTTTTTATATATTATAATCATAATTTTGATATATCAAAATAGGAGATTTGAA-3′
Tdtrop-B	5′-TCGATTCAAATCTCCTATTTTGATATATCAAAATTATGATTATAATATATAAAAAAATAAAGAAAATGTCAAGCTGAATCCC-3′
Tptrop-A	5′-GATCTACTGAAAAATTGACGTTTCCTGCGTGATCCGCTACTTTGATGCATCAAAATTCACCCAAGGAGTGTCAC-3′
Tptrop-B	5′-TCGAGTGACACTCCTTGGGTGAATTTTGATGCATCAAAGTAGCGGATCACGCAGGAAACGTCAATTTTTCAGTA-3′
Tdtrop-mutF3	5′-GATCGGGATTCAGCTTGACATTTTCTTTATTTTTTTATATATTATAATCATAA**GC**TT**C**ATAT**T**TCAAAATAGGAGATTTGAA-3′
Tdtrop-mutR3	5′-TCGATTCAAATCTCCTATTTTGAAATATGAAGCTTATGATTATAATATATAAAAAAATAAAGAAAATGTCAAGCTGAATCCC-3′
qRT-PCR primers and probes	
troA-F1	5′-GCATGGTTGCCGACATAGC-3′
troA-R1	5′-CCCGCACCCATAAGAGCTT-3′
troA-P1	FAM-CACATTAACTTCATCTCCGCCGACAACTTT-TAMRA
flaA-F1	5′-TGACGGCTGGAGAGAATTGG-3′
flaA-R1	5′-GGATAAAGCCTCAATTCCCTAGACT-3′
flaA-P1	FAM-ATGGAACAATCCTTCATACATTGCGAACGT-TAMRA

FAM, 6-carboxyflourescein; TAMRA, 5-carboxytetramethylrhodamin.

### TroR_Td_ is a Mn^*2+*^- and Fe^*2+*^-dependent repressor

**Fig. 4:**
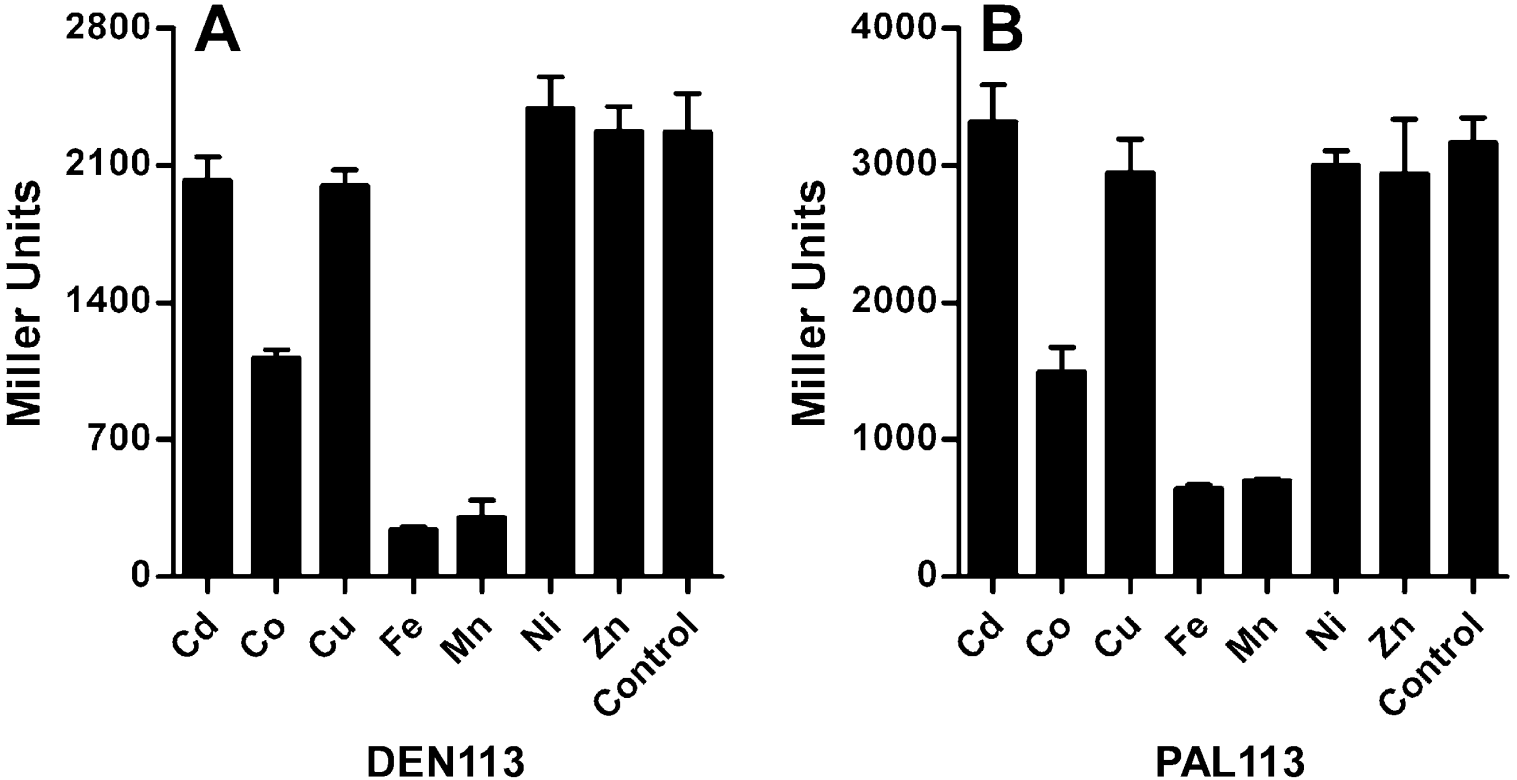
The TroR_Td_ repressor responds to Mn^2+^ and Fe^2+^ in *E. coli*. (A) Strain DEN113 expressing pTDE100 (*T. denticola tro*P/O-*lacZ* reporter) and pPJB113 (expresses TroR_Td_) or (B) strain PAL113 expressing pPAL100 (*T. pallidum tro*P/O-*lacZ* reporter) and pPJB113 (expresses TroR_Td_) were grown aerobically overnight in M9CG media supplemented with various divalent cations (5 μM) or in unsupplemented M9CG media (control). β-Galactosidase activity of the overnight cultures was determined as described by Miller (1972). Results represent the means and standard deviations of three independent experiments.

TroR_Tp_ has previously been shown to be a Mn^2+^-dependent repressor (Posey *et al*., 1999). In order to determine the metal responsiveness of TroR_Td_ expressed in *E. coli*, we measured β-galactosidase activity in strains DEN113 and PAL113 in response to different metal ions. Both strains were grown in M9CG individually supplemented with 5 μM Cd^2+^, Co^2+^, Cu^2+^, Fe^2+^, Mn^2+^, Ni^2+^ and Zn^2+^. Addition of either Mn^2+^ or Fe^2+^ resulted in substantially decreased β-galactosidase activity from both DEN113 and PAL113, indicating that TroR_Td_ repressor function was primarily responsive to these two metals (Fig. 4A and B). Similarly, the addition of Co^2+^ partially repressed promoter activity; however, no effect was observed upon addition of Cd^2+^, Ni^2+^, Zn^2+^, or in the absence of metals (Fig. 4A and B). These results are in agreement with previous reports demonstrating that TroR_Tp_ responds to Mn^2+^ in *E. coli* (Posey *et al*., 1999; Hazlett *et al*., 2003). However, our results demonstrated that TroR_Td_ also appears to respond to Fe^2+^. This observation is consistent with other members of the DtxR family that are primarily known to be Fe-responsive. In contrast to TroR_Tp_ that has been reported to be Zn^2+^-responsive (Hazlett *et al*., 2003), TroR_Td_ did not repress promoter activity in response to Zn^2+^ supplementation. Taken together, these findings suggest that TroR_Td_ is unique in its metal-sensing capacities in comparison with TroR_Tp_, and shares properties with both MntR- and DtxR-type metalloregulators.

### *T. denticola tro* operon is negatively regulated by Mn^*2+*^ and Fe^*2+*^

**Fig. 5:**
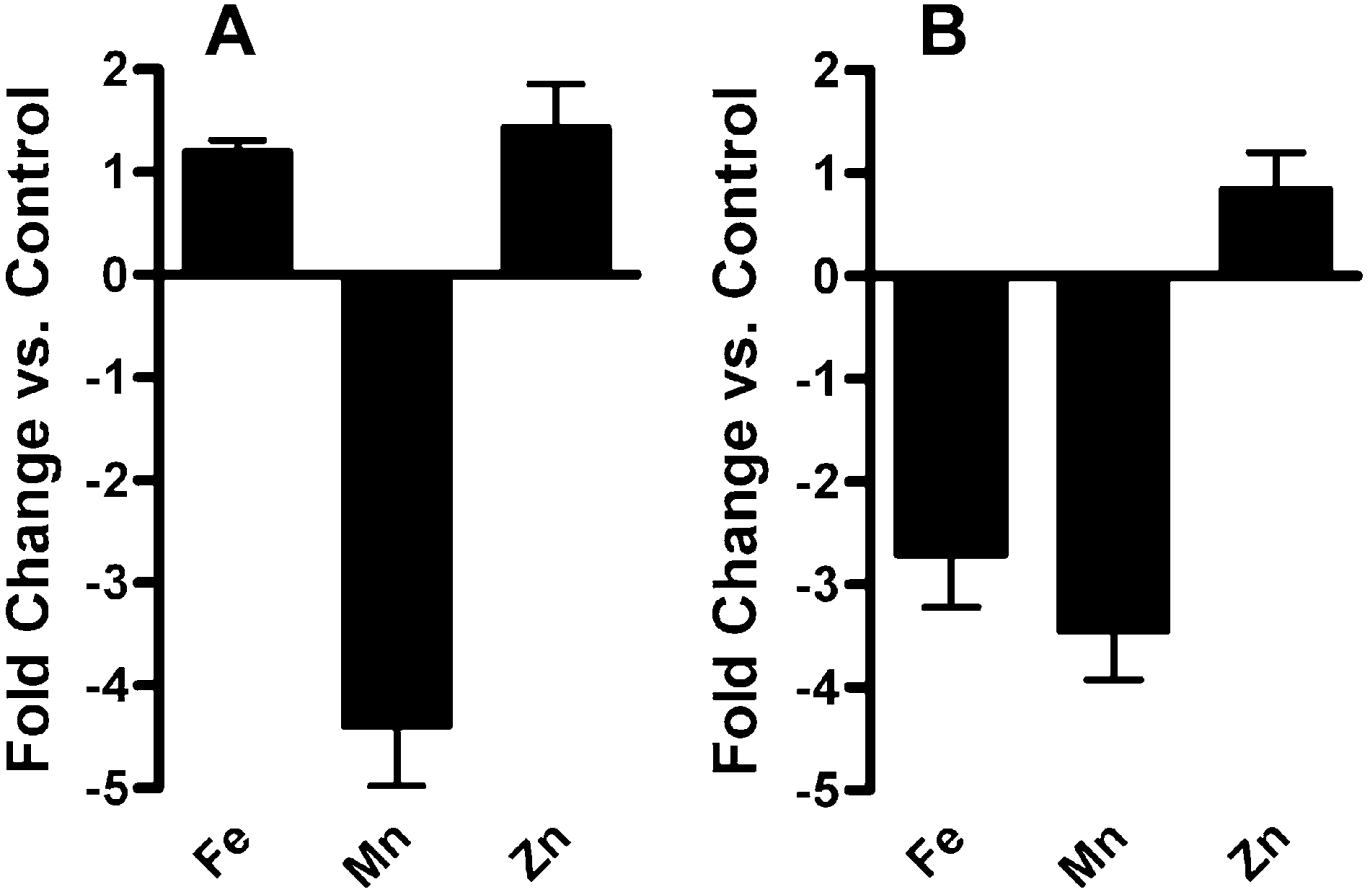
*T. denticola tro* operon is negatively regulated by Mn^2+^ and Fe^2+^. Expression of *troA* was analysed by qRT-PCR. RNA extracted from *T. denticola* grown in NOS-EC media supplemented with 5 μM Fe^2+^, Mn^2+^ or Zn^2+^ was quantified at (A) 24 h or (B) 48 h post inoculation using specific primers and probes with the Taqman system. Values have been normalized to the internal control, *flaA*. Fold changes are relative to spirochaetes grown in NOS-EC media lacking metal supplmentation (control). Results represent the means and standard deviations of three independent experiments performed in quadruplicate.

To determine the response of the *T. denticola tro* operon to specific cations, spirochaetes were grown in NOS-EC media (chelated NOS media with EX-CYTE) supplemented with 5 μM Fe^2+^, Mn^2+^ or Zn^2+^. At 24 and 48 h post inoculation, RNA was harvested and *troA* transcript levels were quantified by qRT-PCR. Relative amounts of the *troA* transcripts from spirochaetes grown in the presence of cations were then compared with transcript levels from unsupplemented controls. All reactions were normalized to *flaA* as an internal control (Frederick *et al*., 2008). At 24 h, a fourfold decrease in *troA* transcript levels was observed in the presence of Mn^2+^ while *troA* expression from cultures supplemented with Fe^2+^ or Zn^2+^ were similar to control levels (Fig. 5A). Interestingly, at 48 h, a three- to fourfold decrease in *troA* transcript levels was observed in the presence of Fe^2+^ and Mn^2+^ respectively, while expression from cultures supplemented with Zn^2+^ remained unchanged (Fig. 5A). Based upon these results and consistent with the *E. coli lacZ*-transcriptional fusion assays, it appears that the *T. denticola tro* operon is negatively regulated by Mn^2+^ and Fe^2+^, but not Zn^2+^.

### TroR_Td_ proteins lacking the SH3-like domain demonstrate decreased repression of *T. denticola* P/O_tro_-*lacZ*, but not *T. pallidum* P/O_tro_-*lacZ*

**Fig. 6:**
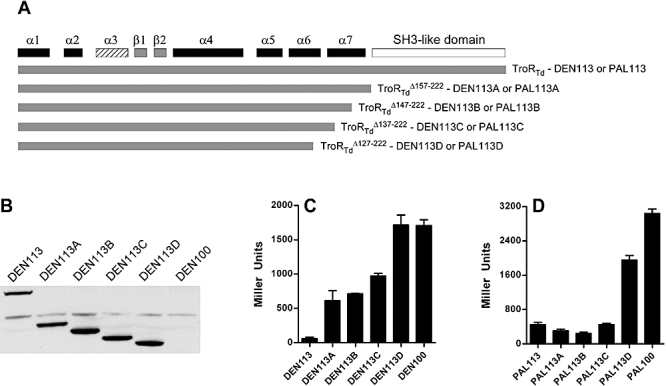
TroR_Td_ deletion mutants exhibit variable repressor activity in *E. coli*. A. Physical maps of wild type and C-terminally truncated TroR_Td_ proteins. TroR_Td_ proteins are represented as grey lines and amino acid deletions are indicated. Names of the strains expressing the various TroR_Td_ deletion constructs in the presence of pTDE100 (*T. denticola tro*P/O-*lacZ* reporter) or pPAL100 (*T. pallidum tro*P/O-*lacZ* reporter) are also indicated. DtxR secondary structure is indicated: α-helices (α1, 2, 4, 5, 6) are shown in black, the DNA recognition helix (α3) is shown as a hatched line, β-strands (β1–2) are shown in grey, the proline-containing tether region (α7) is shown in black and SH3-like domain is shown in white. Full-length and mutant *troR* alleles were PCR-amplified from *T. denticola* ATCC 35405 chromosomal DNA and cloned into the NcoI and HindIII restriction sites of pBAD/HisA to facilitate the expression of TroR_Td_ proteins. B. Immunoblot analysis of full-length and truncated TroR_Td_ proteins expressed by: DEN113, DEN113A, DEN113B, DEN113C, DEN113D and DEN100. Effect of C-terminal deletion mutations on the expression of (C) pTDE100 or (D) pTPA100 reporter constructs. *E. coli* TOP10 cells harbouring both a TroR_Td_ expression construct and a *tro*-P/O-*lacZ* reporter plasmid were grown aerobically overnight in LBL media. β-Galactosidase activity of the 12 h cultures was determined as described by Miller (1972). Results represent the means and standard deviations of three independent experiments.

Both TroR_Tp_ and *B. subtilis* MntR lack the C-terminal regions present in many members of the DtxR family of metalloregulators; however, both demonstrate repressor activity in the absence of this domain. In order to assess the importance of the TroR_Td_ C-terminal SH3-like domain with respect to repressor activity, full-length and truncated *troR* alleles were cloned into pBAD/HisA and expressed in *E. coli*. A schematic representing the C-terminal TroR_Td_ truncations is shown in Fig. 6A. Immunoblot analysis demonstrated that all of the truncated proteins were expressed in *E. coli* at the expected sizes and at similar levels (Fig. 6B). Removal of the C-terminal region of TroR_Td_ did not improve solubility of the recombinant proteins (data not shown). β-Galactosidase assays were conducted in *E. coli* strains harbouring the *tro*-P/O *lacZ*-transcriptional fusion constructs, pTDE100 or pTPA100, along with plasmids expressing full-length TroR_Td_ (strain DEN113) or various truncated derivatives as follows: TroR_Td_^Δ157−222^ (strain DEN113A), TroR_Td_^Δ147−222^ (strain DEN113B), TroR_Td_^Δ137−222^ (strain DEN113C) and TroR_Td_^Δ127−222^ (strain DEN113D). Results for the *T. denticola tro*-P/O region demonstrated significantly decreased β-galactosidase activity in strain DEN113 (96% repression), compared with the control strain DEN100, as expected. In contrast, expression of the truncated TroR_Td_ proteins in strains DEN113A, DEN113B and DEN113C demonstrated increased β-galactosidase activity, corresponding to 64%, 58% and 43% repression respectively, indicating decreased repressor function. Expression of TroR_Td_^Δ127−222^, a deletion extending into the proline-containing region of the protein, in strain DEN113D resulted in maximal β-galactosidase activity (similar to DEN100), indicating virtually complete loss of repressor function (Fig. 6C). These results suggest that the TroR_Td_ SH3-like domain is important for repressor activity and presumably binding to the *T. denticola tro*-P/O region.

Transcriptional fusion analyses with the *T. pallidum tro*-P/O region in *E. coli* revealed that expression of full-length TroR_Td_, TroR_Td_^Δ157−222^, TroR_Td_^Δ147−222^ or TroR_Td_^Δ137−222^ resulted in maximal repressor activity. As shown in Fig. 6D, expression of full-length TroR_Td_ in strain, PAL113 resulted in decreased β-galactosidase activity corresponding to 85% repression compared with control strain PAL100. Similarly, β-galactosidase activity decreased in strains PAL113A, PAL113B and PAL113C (90%, 92% and 85% repression respectively), indicating intact repressor function. In contrast, TroR_Td_^Δ127−222^ expression in strain PAL113D resulted in only slightly decreased β-galactosidase activity corresponding to partial repressor activity (36%) (Fig. 6D). Taken together, these results suggest that the SH3-like domain of TroR_Td_ is required for maximal repression of the *T. denticola tro*-P/O; however, this C-terminal extension does not appear to be critical for repression of the *T. pallidum tro*-P/O. In addition, it is possible that the differences observed in the positions of the −10 and −35 sequences within the *T. denticola* and *T. pallidum tro*-P/O regions may affect repressor binding.

### *T. denticola* TroR_Td_^Δ157−222^ maintains metal specificity

**Fig. 7:**
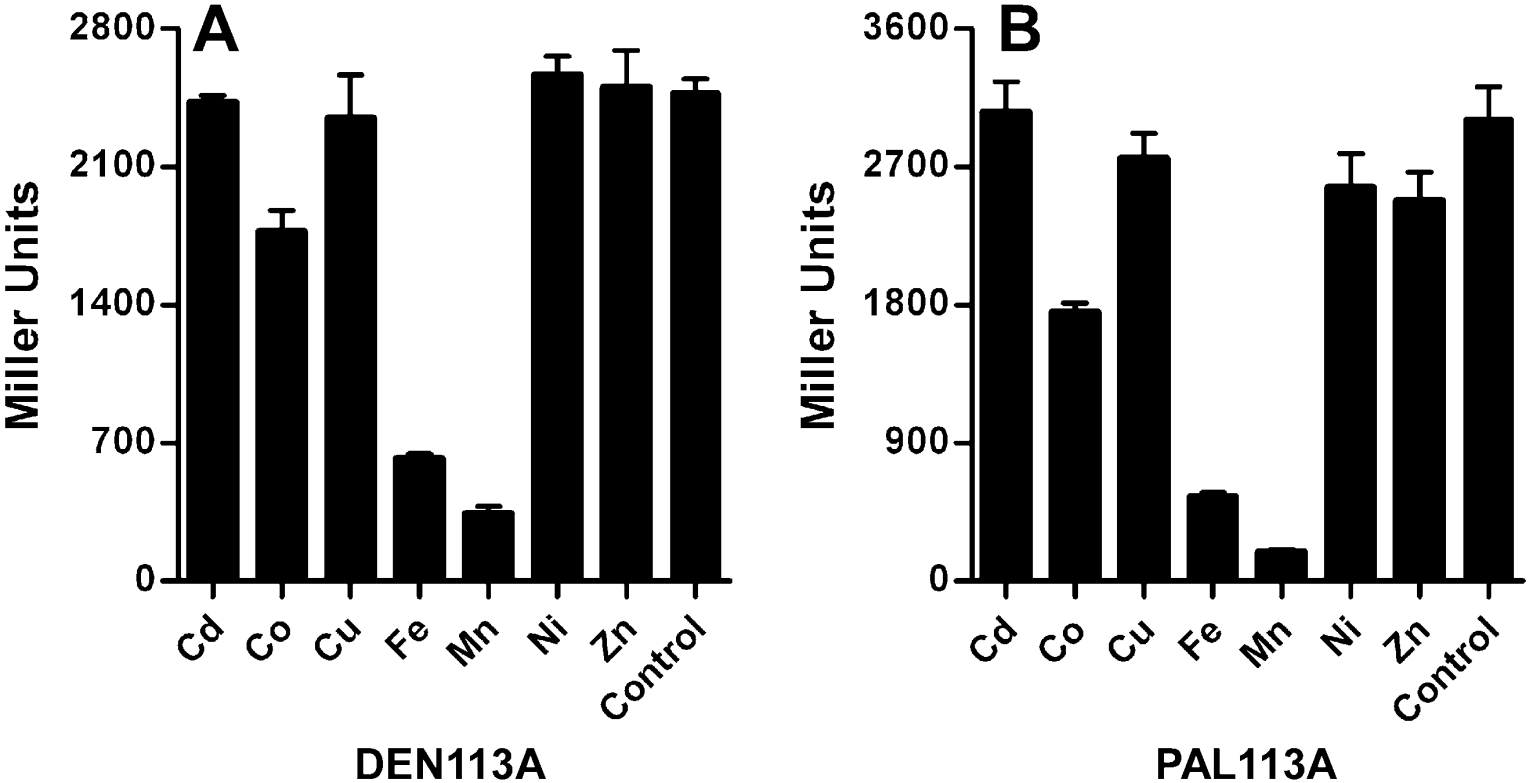
TroR_Td_^Δ157−222^ repressor responds to Mn^2+^ and Fe^2+^ in *E. coli*. (A) Strain DEN113A harbouring pTDE100 (*T. denticola tro*P/O-*lacZ* reporter) and pPJB113A (expresses TroR_Td_^Δ157−222^) or (B) strain PAL113A harbouring pPAL100 (*T. pallidum tro*P/O-*lacZ* reporter) and pPJB113A (expresses TroR_Td_^Δ157−222^) were grown aerobically overnight in M9CG assay media supplemented with various divalent cations (5 μM) or in unsupplemented M9CG media (control). β-Galactosidase activity of the overnight cultures was determined as described by Miller (1972). Results represent the means and standard deviations of three independent experiments.

Unlike other members of the DtxR family of metalloregulators, both TroR_Tp_ and *B. subtilis* MntR are manganese-responsive and lack C-terminal SH3-like domains. In order to assess the importance of the TroR_Td_ C-terminal domain with respect to metal specificity, β-galactosidase activity from strains DEN113A and PAL113A was measured in response to metal ion supplementation. In the presence of TroR_Td_^Δ157−222^ expression, β-galactosidase activity from both DEN113A and PAL113A decreased in response to Mn^2+^ and Fe^2+^ and also decreased slightly in response to Co^2+^ (Fig. 7A and B). β-Galactosidase activity remained unchanged in the presence of Cd^2+^, Cu^2+^, Ni^2+^ and Zn^2+^ (Fig. 7A and B). These results indicated that similar to full-length TroR_Td_, TroR_Td_^Δ157−222^ maintained repressor activity in the presence of Mn^2+^, Fe^2+^ and Co^2+^. Based on these results, it appears that the C-terminal domain of TroR_Td_ is not important for metal specificity and the role of this region of the protein in metal binding, if any, remains unclear.

## Discussion

As intracellular concentrations of both essential and nonessential metals can be toxic to bacterial cells, their uptake is tightly regulated by metal-dependent, transcriptional repressor proteins (Outten and O'Halloran, 2001; Patzer and Hantke, 2001; Glasfeld *et al*., 2003). These regulatory proteins (e.g. DtxR and Fur families) serve as direct links between intracellular metal levels and the expression of appropriate uptake, efflux and storage systems. In general, metal cations bind reversibly to a metal binding site altering the conformation of the repressor to effect changes in gene expression. The affinity of metal ions for this site serves to maintain the intracellular concentration of metals within strict, biologically acceptable limits in a cell. The selectivity of the site is essential to ensure that other metals do not interfere with these homeostasis mechanisms (Guedon and Helmann, 2003). In addition to mediating physiological responses to environmental levels of metals, it is well established that metal-dependent repressors, such as DtxR and Fur, modulate the virulence phenotypes of a number of important human pathogens (Jakubovics and Jenkinson, 2001; Guedon and Helmann, 2003).

DxtR and its homologues represent a distinct family of metal-dependent repressors that primarily regulate the expression of genes involved in both metal uptake and virulence. In *Corynebacterium diphtheria*, genes encoding iron transport proteins are negatively regulated by DtxR (Tao *et al*., 1994; Schmitt, 1999). As intracellular concentrations of ferrous iron increase, DtxR becomes iron-loaded, dimerize, and binds to a palindromic target sequence within DtxR-regulated promoters, repressing the transcription of downstream genes (Qiu *et al*., 1995; Tao *et al*., 1995). The X-ray crystal structure of DtxR has been solved and has led to a better understanding of the interaction of DtxR with its metal ligands (Qiu *et al*., 1995; D'Aquino *et al*., 2007). Apo-DtxR contains two metal binding sites: binding site 1, which co-ordinates a metal between H79, E83 and H98 (C-terminal SH3 domain residues E170 and Q173 may also contribute), is considered an auxiliary binding site that may act cooperatively to enhance binding of a second metal ion to the primary binding site, binding site 2 (Qiu *et al*., 1995; Pennella and Giedroc, 2005; D'Aquino *et al*., 2007). This binding site is required for metal-dependent DtxR repressor activity and co-ordinates the metal between M10, C102, E105 and H106. While Ni^2+^, Co^2+^ and Mn^2+^ have been shown to activate DtxR *in vitro*, Fe^2+^ is the specific activator *in vivo* (Ikeda *et al*., 2005; Pennella and Giedroc, 2005).

A much more distant member of the family is MntR, a DtxR homologue from *B. subtilis* that has been shown to be selective for Mn^2+^ and regulates the expression of two manganese transporters under low Mn^2+^ conditions (Glasfeld *et al*., 2003; Kehres and Maguire, 2003; Pennella and Giedroc, 2005). Unlike DtxR, MntR binds Mn^2+^ in a binuclear manganese cluster, in which two metal ions are co-ordinated between D8, E11, H77, E99, E102 and H103 (see Fig. 2) This unique configuration confers selectivity for Mn^2+^ over Fe^2+^. Recent studies have demonstrated that the D8 residue confers some of the Mn^2+^ specificity in MntR, as changing that amino acid to M8 (DtxR-like) relaxes the specificity for Mn^2+^ (Guedon and Helmann, 2003; Golynskiy *et al*., 2005). While DtxR and MntR have some key active site amino acid residues in common (e.g. H79, E105 and H106), MntR lacks the SH3-like domain, has different amino acids in key metal binding site residues and functions in a different cytosolic environment so that the two proteins function/respond quite differently *in vivo* (Guedon and Helmann, 2003).

Other DtxR family members include the Fe- and Mn^2+^-responsive repressors group A *Streptococcus* MtsR and *S. epidermidis* SirR. Both MtsR and SirR have been shown to possess the majority of the conserved metal co-ordination sites identified in DtxR and are predicted to have similar structural domains (Hill *et al*., 1998; Bates *et al*., 2005). SirR, however, is most similar (38%) at the amino acid level to *T. pallidum* TroR (Hill *et al*., 1998), a distinct member of this group that has been identified and partially characterized. TroR_Tp_ has structural features that suggest that it may represent a functional intermediate of the Fe^2+^-dependent DtxR and the Mn^2+^-dependent MntR (Hardham *et al*., 1997; Posey *et al*., 1999; Hazlett *et al*., 2003). For example, TroR_Tp_ is similar to MntR in overall protein structure as both proteins lack the characteristic DtxR SH3-like domain and have some amino acid residues that correspond to key residues involved in MntR's binuclear Mn^2+^ metal pocket (see Fig. 2, shaded amino acids). Interestingly, TroR_Tp_ also has residues that correspond to key residues in DtxR sites 1 and 2 (see Fig. 2, shaded amino acids). Experimentally, TroR_Tp_ has been shown to be Mn^2+^-specific when assayed using purified protein in gel mobility shift assays using the *tro* operon-P/O as a target sequence (Posey *et al*., 1999). In contrast, Hazlett *et al*. (2003) showed that recombinant TroR_Tp_ responds more efficiently to Zn^2+^ than Mn^2+^, using *tro*P/O-*lacZ* reporter constructs in *E. coli*. Fe^2+^ did not affect TroR_Tp_ binding to the Tro P/O in either assay system. It is interesting to note that while TroR_Tp_ effectively regulates the Mn/Zn/Fe TroABCD transport system (Posey *et al*., 1999; Hazlett *et al*., 2003), it does not appear to regulate the specific, high-affinity Zn^2+^ uptake system (Znu operon) in *T. pallidum* (Desrosiers *et al*., 2007)*.* As these studies on TroR_Tp_ were done in different strains and genetic backgrounds, it is difficult to determine the exact metal specificity of this protein. Therefore, the exact metal specificity of TroR_Tp_ in *T. pallidum* remains in question.

In the present study, we identified the *troABCDR* operon in the *T. denticola* ATCC 35405 genome and characterized the activity of TroR_Td_ as a metal-dependent repressor that responded primarily to Mn^2+^ and Fe^2+^. Based on previous studies with TroR_Tp_, we predicted that TroR_Td_ would bind to the putative TroR binding sequence upstream of the *T. denticola tro* operon to repress expression of these genes. Our results demonstrated that TroR_Td_ repressed transcription of both *T. denticola* and *T. pallidum tro*-P/O *lacZ* reporter gene fusions in *E. coli*. In addition, mutation of the putative TroR_Td_ binding sequence upstream of *troA* resulted in a considerable decrease in repressor activity. These findings provide evidence that TroR_Td_ is a negative regulator of the *tro* operon. Interestingly, significant repression was demonstrated in response to Mn^2+^ or Fe^2+^, and partial repression was observed in response to Co^2+^ while Zn^2+^ had no effect on gene expression. Consistent with these findings, qRT-PCR analyses conducted with *T. denticola* grown under metal-deplete or -replete conditions demonstrated that *troA* expression decreased in the presence of Mn^2+^ and Fe^2+^, but not Zn^2+^. Taken together these results suggest that TroR_Td_ has different metal specificity than TroR_Tp_ (Mn^2+^ or Zn^2+^) or MntR (Mn^2+^) and may be more similar to DtxR, SirR and MtsR.

Previous studies by Guedon and Helmann (2003) have demonstrated that, as important as the active site residues are in metal specificity, the cytosolic metal environment affects the metal-dependent response of a particular repressor in different host backgrounds. When they ‘converted’ DtxR to a Mn-dependent repressor by introducing M10D and C102E (MntR-like) and expressed the mutant protein in *B. subtilis*, DtxR (M10D/C102E) as predicted responded to Mn^2+^. However, when intracellular concentrations of Fe^2+^ were increased by deregulating iron uptake in a *B. subtilis fur* mutant, DtxR (M10D/C102E) also responded to Fe^2+^ (Guedon and Helmann, 2003). Thus, they showed that while key residues within the binding site affect metal specificity, the concentration of specific metals within a particular cell also influence the regulatory capacity of a given metal-dependent repressor protein. Interestingly, Posey and Gherardini (2000) have measured and compared the intracellular levels of Mn^2+^ (0.79 and 0.24 nmol mg^−1^ of protein respectively) and Fe^2+^ (4.2 and 3.5 nmol mg^−1^ of protein respectively) in *E. coli* and *T. denticola*. While there are some differences in the intracellular levels of these metals (particularly Mn) under the conditions tested, it is interesting to note that the data presented in this report, as measured in *E. coli* (*lacZ* reporter assays) and *T. denticola* (qRT-PCR), indicate that TroR_Td_ responded similarly to Mn^2+^ and Fe^2+^ in both cytosolic backgrounds*.*

Secondary structure predictions and amino acid comparisons of TroR_Td_ with DxtR, MntR and TroR_Tp_ revealed the presence of conserved N-terminal helical domains as well as conserved metal binding site residues. Additionally, TroR_Td_ had a C-terminal SH3-like domain. The importance of the C-terminal extension present on TroR_Td_ was investigated using a number of C-terminally truncated TroR proteins. Our results demonstrated that loss of the C-terminal region of TroR_Td_ affected repressor function at the *T. denticola tro*-P/O, but not at the *T. pallidum tro*-P/O. These results are consistent with the fact that TroR_Tp_ naturally lacks this C-terminal extension. It is possible that the differences observed in the *tro*-P/O regions of these two treponemes may reflect their different-sized TroRs; i.e. each species has optimized its P/O region for TroR binding. Conversely, the different-length TroRs may be produced to compensate for the altered *tro*-P/O regions. In any case, it appears that the TroR_Td_ requires an extended C-terminal region for maximal repression of the *T. denticola tro*-P/O. Further results indicated that the proline-containing region may be necessary for TroR to function at both *T. denticola* and *T. pallidum tro*-P/O regions as a deletion affecting this region-abrogated repression. This domain may function in protein–protein interactions that may be important for subsequent DNA binding; however, this remains to be experimentally determined. The metal specificity of C-terminally truncated TroR remained unchanged, suggesting that this domain was not involved in metal binding. Similar to DtxR, the specific function of TroR_Td_ C-terminal domain remains unclear.

In summary, we have demonstrated that TroR_Td_ is a Mn^2+^- and Fe^2+^-dependent transcriptional regulator that negatively regulates expression of the *T. denticola tro* operon. Structural predictions show that TroR_Td_ possesses conserved metal binding site residues as well as an SH3-like domain. Overall, these findings suggest that TroR_Td_ is a novel member of the DtxR family of transcriptional regulators and would be predicted to play a critical role in regulating Mn^2+^ and Fe^2+^ levels in *T. denticola*. Further studies will be required to determine the role of the *T. denticola tro* operon in metal ion homeostasis in natural infections. Studies are ongoing to obtain soluble TroR for further biochemical and crystallography analyses to gain further insight into the structure and function of TroR_Td_.

## Experimental procedures

### Bacterial strains, plasmids, chemical reagents and growth conditions

The bacterial strains and plasmids used in this study are described in Table 3. Puratronic metals were obtained from Alfa Aesar: Cd as Cadmium sulphate, 99.999%; Co as Cobalt (II) sulphate 99.999%; Cu as Copper (II) sulphate, 99.999%; Fe as Iron (II) sulphate, 99.999%; Mn as Manganese (II) sulphate, 99.999%; Ni as Nickel (II) sulphate, 99.9985%; Zn as Zinc sulphate, 99.999%; Magnesium sulphate, 99.997%; Calcium chloride, 99.9965%. Tris(2-carboxyethyl)-phosphine hydrochloride (TCEP) was obtained from Pierce. All other chemicals were obtained from Sigma. *E. coli* strains were grown at 37°C on Difco LB-Lennox (LBL) agar or in LBL broth unless otherwise stated. For metal specificity assays, *E. coli* strains were grown in Difco M9 Minimal Salts supplemented with 0.5% w/v Bacto Casamino Acids and 0.2% v/v glycerol (M9CG). When appropriate, antibiotics were used at the following concentrations: 100 μg ml^−1^ ampicillin, 25 μg ml^−1^ chloramphenicol and 25 μg ml^−1^ kanamycin (Km).

**Table 3:** Bacterial strains and plasmids used in this study.

Strain or plasmid	Description	Reference or source
Strain		
*T. denticola*		
ATCC 35405	Type strain; human oral isolate	Chan *et al*. (1993)
*E. coli*		
TOP10	High-efficiency transformation: Ap^s^, Cm^s^, Km^s^	Invitrogen
LYS114	TOP10 (pLysE + pPJB114): Ap^r^, Cm^r^	This study
DEN100	TOP10 (pTDE100 + pBAD/HisA): Ap^r^, Km^r^	This study
DEN113	TOP10 (pTDE100 + pPJB113): Ap^r^, Km^r^	This study
DEN103	TOP10 (pTDE103 + pBAD/HisA): Ap^r^, Km^r^	This study
DEN103R	TOP10 (pTDE103 + pPJB113): Ap^r^, Km^r^	This study
DEN113A	TOP10 (pTDE100 + pPJB113A): Ap^r^, Km^r^	This study
DEN113B	TOP10 (pTDE100 + pPJB113B): Ap^r^, Km^r^	This study
DEN113C	TOP10 (pTDE100 + pPJB113C): Ap^r^, Km^r^	This study
DEN113D	TOP10 (pTDE100 + pPJB113D): Ap^r^, Km^r^	This study
PAL100	TOP10 (pTPA100 + pBAD/HisA): Ap^r^, Km^r^	This study
PAL113	TOP10 (pTPA100 + pPJB113): Ap^r^, Km^r^	This study
PAL113A	TOP10 (pTPA100 + pPJB113A): Ap^r^, Km^r^	This study
PAL113B	TOP10 (pTPA100 + pPJB113B): Ap^r^, Km^r^	This study
PAL13C	TOP10 (pTPA100 + pPJB113C): Ap^r^, Km^r^	This study
PAL113D	TOP10 (pTPA100 + pPJB113D): Ap^r^, Km^r^	This study
Plasmids		
pACYC184	Cloning vector; p15A ori: Cm^r^, Tc^r^	New England Biolabs
pBAD/His/*lacZ*	Arabinose inducible LacZ expression vector; pMB1 ori: Ap^r^	Invitrogen
pUni/V5-His A	Echo Cloning vector; R6K ori: Km^r^	Invitrogen
pBAD/His A	Arabinose inducible expression vector; pMB ori: Ap^r^	Invitrogen
pLysE	Constitutively expresses T7 lysozyme; p15A ori: Cm^r^	Invitrogen
pPBMB100	Promoterless *lacZ* reporter vector; p15A ori: Cm^r^	This study
pPBMB101	Promoterless *lacZ* reporter vector; p15A ori: Km^r^	This study
pTPA100	*T. pallidum tro*P/O-*lacZ* reporter construct; pPBMB101 derivative: Km^r^	This study
pTDE100	*T. denticola tro*P/O-*lacZ* reporter construct; pPBMB101 derivative: Km^r^	This study
pTDE103	*T. denticola tro*P/O-*lacZ* reporter construct; mutated operator sequence; pPBMB101 derivative: Km^r^	This study
pPJB113	TroR_Td_ expression construct; pBAD/His A derivative: Ap^r^	This study
pPJB113A	TroR_Td_^Δ157−222^ expression construct; pBAD/His A derivative: Ap^r^	This study
pPJB113B	TroR_Td_^Δ147−222^ expression construct; pBAD/His A derivative: Ap^r^	This study
pPJB113C	TroR_Td_^Δ137−222^ expression construct; pBAD/His A derivative: Ap^r^	This study
pPJB113D	TroR_Td_^Δ127−222^ expression construct; pBAD/His A derivative: Ap^r^	This study
pPJB114	TroR_Td_-6×His expression construct; pBAD/His A derivative: Ap^r^	This study

*Treponema denticola* ATCC 35405 was grown at 37°C under anaerobic conditions (4% H_2_, 5% CO_2_, 91% N2) in NOS media (ATCC medium 1494) supplemented with 1% EX-CYTE (Millipore; NOS-E media) instead of rabbit serum. Growth rates in NOS-E media were found to be similar to those in NOS media. Metal-deplete NOS-E media (NOS-EC media) was prepared by chelating NOS-E media two times with 2.5% (w/v) Chelex 100 under anaerobic conditions. NOS-EC media was then supplemented with 1 mM MgSO_4_ and 0.1 mM CaCl_2_ and filter-sterilized.

### Recombinant DNA techniques

DNA manipulations were performed using standard methods. PCR was performed using the Expand High Fidelity PCR System (Roche Applied Science). PCR and restriction enzyme-digested products were purified using a QIAquick Gel Extraction Kit (Qiagen). Plasmids were purified using a QIAprep Spin Miniprep Kit (Qiagen). Ligation reactions were performed using a Fast-Link Quick Ligase Kit (Epicentre Technologies). Chemically competent *E. coli* TOP 10 cells were transformed as per manufacturer's instructions (Invitrogen). DNA sequencing was performed by ACGT.

### Construction of promoterless *lacZ* reporter vectors

A 1.96 kb fragment harbouring the p15A origin of replication and chloramphenicol-resistance marker from pACYC184 in addition to a 3.38 kb fragment containing the *lacZ* locus from pBAD/His/*lacZ* were PCR-amplified using the OriCm-F1/OriCm-R1 and LacZ-F2/LacZ-R2 primer pairs respectively (the PstI and BglII sites in the linker regions are underlined; Table 2). The amplified products were then restriction-digested and ligated to one another to produce pPBMB100. A 0.80 kb fragment containing the kanamycin-resistance marker from pUni/V5-His A was subsequently amplified via PCR using the Km-FNh/Km-RNc primer pair (the BsaI/NheI and BsaI/NcoI sites in the linker regions are underlined; Table 2) and cloned into pPBMB100 digested with NheI and NcoI to yield pPBMB101.

### Construction of *troP*/O-*lacZ* reporter vectors

Complementary oligonucleotides (Tdtrop-A/Tdtrop-B and Tptrop-A/Tptrop-B) encoding the *T. denticola* and *T. pallidum tro-*P/O regions were mixed at concentrations of 5 μM each, heated to 95°C for 5 min and then allowed to cool to room temperature (RT) to facilitate annealing (the 5′-BamHI and 3′-XhoI 4 bp overhangs incorporated into the ends of oligonucleotides are underlined; Table 2). The resulting double-stranded DNA fragments were then cloned into pPBMB101 digested with BamHI and XhoI yielding pTDE100 and pTPA100 respectively (Table 3). Complementary oligonucleotides (Tdtrop-mutF3/Tdtrop-mutR3) encoding the *T. denticola tro*-P/O with four changes in the predicted operator sequence (see bolded bases in Table 2) were annealed and cloned as described above resulting in pTDE103. Constructs were confirmed by sequencing.

### Construction of TroR_Td_ expression vectors

Full-length, truncated and 6×His-tagged *troR* alleles were amplified from frozen stocks of *T. denticola* ATCC 35405 using the following primer pairs: TdtroR-F2/TdtroR-R3, TdtroR-F2/TdtroR-RA, TdtroR-F2/TdtroR-RB, TdtroR-F2/TdtroR-RC, TdtroR-F2/TdtroR-RD and TdtroR-F2/TdtroR-RH2 (the BsmBI/NcoI and BsmBI/HindIII sites in the linker regions are underlined; Table 2). The amplified products were then cloned into pBAD/HisA digested with NcoI and HindIII yielding pPJB113, pPJB113A, pPJB113B, pPJB113C, pPJB113D and pPJB114 respectively (Table 3). Constructs were confirmed by sequencing.

### β-Galactosidase assays

Combinations of the TroR_Td_ expression and *lacZ* reporter constructs were cotransformed into *E. coli* Top10 cells (Table 3). In order to assay promoter activities under metal-rich conditions, cotransformants were inoculated (1/3000) into LBL broth plus antibiotics from overnight cultures and grown for 12 h at 37°C with shaking. In order to assay promoter activities under metal-deplete conditions, cotransformants were inoculated (1/3000) into M9CG plus antibiotics supplemented with 5 μM Cd^2+^, Co^2+^, Cu^2+^, Fe^2+^, Mn^2+^, Ni^2+^ or Zn^2+^ from overnight cultures and grown for 12 h at 37°C with shaking. TroR_Td_ expression was found to be sufficiently leaky from the *ara*BAD promoters of pPJB113, pPJB113A, pPJB113B, pPJB113C, pPJB113D during these assays and did not warrant the use of l-arabinose as an inducer. β-Galactosidase assays were performed as previously described by Miller (1972).

### *T. denticola* RNA purification, cDNA synthesis and qRT-PCR

For metal regulation assays, *T. denticola* was grown to late log phase in NOS-E media, pelleted by centrifugation (2800 *g*, 10 min) and then re-suspended in NOS-EC media. NOS-EC media supplemented with 5 μM Fe^2+^, Mn^2+^ or Zn^2+^ was inoculated with ∼10^7^ spirochaetes ml^−1^ and RNA was harvested at 24 and 48 h. qRT-PCR experiments were performed as previously described for *Borrelia burgdorferi* (Burtnick *et al*., 2007). Briefly, *T. denticola* RNA was extracted using TRI-Reagent (Sigma) as described by the manufacturer. RNA was treated with RNase-free DNase (Qiagen) and purified using the RNeasy miniprep kit (Qiagen). SuperScript III (Invitrogen) was used to synthesize first-strand cDNA following manufacturer's instructions. qRT-PCR primers and probes specific for *troA* and *flaA* were designed using Primer Express 1.0 and are shown in Table 2. Reactions were performed in a total volume of 20 μl using TaqMan Universal PCR Master Mix (Applied Biosystems, Foster City, CA), 1–2 ng of first-strand cDNA, 300 nm forward and reverse primers and 250 nm probe. All reactions were carried out on the ABI PRISM 7900HT Sequence Detection System (Applied BioSystems) using a PCR cycle of 2 min at 50°C, 10 min at 95°C, followed by 40 cycles at 95°C for 15 s and 60°C for 1 min. Each transcript was normalized by comparison with the constitutively expressed, internal control *flaA* (Frederick *et al*., 2008). Three individual assays were performed in quadruplicate. Fold changes were calculated using the ΔΔC_T_ method.

### TroR_Td_ purification and antibody production

For purification of recombinant protein, TroR_Td_-6×His was overexpressed from *E. coli* LYS114 (Table 3). Bacteria were grown in 500 ml of LBL broth plus antibiotics at 37°C with aeration. When the culture reached an OD_600_ of 0.8, protein expression was induced using 0.02% l-arabinose for 2 h. Cells were harvested by centrifugation, re-suspended in Lysis Buffer A (B-PER plus 2 μg ml^−1^ DNase I; Pierce) and incubated for 10 min at RT. The insoluble material was pelleted by centrifugation (27 000 *g*, 15 min, 4°C), re-suspended in Lysis Buffer B (B-PER) and incubated for 10 min at RT. The insoluble material was pelleted, re-suspended in Solubilization Buffer [50 mM Tris (pH 8.0), 50 mM NaCl, 10 mM Imidazole, 0.25 mM TCEP and 0.2% Sarkosyl] and gently agitated for 60 min at 4°C. The remaining insoluble material was removed by centrifugation, the supernatant was filter-sterilized and allowed to batch-bind to HIS-Select Nickel Affinity Gel (Sigma) prior to loading onto a gravity fed column. The flow through was collected and applied to the column a second time, followed by washing of the column with Wash Buffer [50 mM Tris (pH 8.0), 300 mM NaCl, 10% glycerol, 40 mM Imidazole and 0.25 mM TCEP]. Protein was eluted with Elution Buffer [50 mM Tris (pH 8.0), 50 mM NaCl, 10% glycerol, 300 mM Imidazole and 0.25 mM TCEP]. Fractions were analysed by SDS-PAGE and those containing TroR_Td_ were pooled, concentrated and stored in Storage Buffer [20 mM Tris (pH 8.0), 50 mM NaCl, 10% glycerol and 0.25 mM TCEP]. Protein concentrations were determined using a BCA protein assay kit (Pierce). Purified protein was used to raise anti-TroR_Td_ polyclonal antiserum in female New Zealand White rabbits at Cocalico Biologicals using a standard protocol.

### SDS-PAGE and immunoblot analysis

Whole-cell lysates were prepared by pelleting 1 ml each of the *E. coli* cultures and re-suspending the cells in 200 μl of 1× SDS-PAGE sample buffer prior to heating at 100°C for 10 min. Once cooled, 1 μl of DNase (10 000 U ml^−1^; Pierce) was added to each sample to decrease viscosity. Treated lysates were then electrophoresed on 4–20% Express Gels (ISC BioExpress). Proteins were electrophoretically transferred to nitrocellulose membranes (0.45 μm pore size; Invitrogen). Immunoblot analysis was performed as follows at RT: membranes were blocked with TBS-TS [20 mM Tris-HCl (pH 7.5), 500 mM NaCl, 0.1% Tween 20 and 3% skim milk] for 60 min, followed by incubation for 1 h with a 1/2000 dilution of the primary antibody (anti-TroR polyclonal antiserum) in TBS-T [20 mM Tris-HCl (pH 7.5), 500 mM NaCl and 0.1% Tween 20]. Membranes were washed three times with TBS-T, followed by incubation for 1 h with a 1/5000 dilution of the secondary antibody (anti-rabbit IgG-HRP conjugate; Sigma) in TBS-T. Membranes were washed three times with TBS-T and blots were visualized with ECL Plus Western Blotting Detection Reagents (GE Healthcare) as per manufacturer's instructions.
